# A case of labyrinthine pattern basal cell carcinoma^☆^

**DOI:** 10.1016/j.abd.2024.09.009

**Published:** 2025-04-21

**Authors:** Takehiro Nakamura, Toshiyuki Yamamoto

**Affiliations:** Department of Dermatology, Fukushima Medical University School of Medicine, Fukushima, Japan

Dear Editor,

Basal cell carcinoma (BCC) is the most common type of malignant skin tumor and presents with various histopathological subtypes. One of these subtypes exhibits a complex cord-like arrangement of tumor cells, described as a labyrinthine pattern. Reports on BCC with a labyrinthine pattern are primarily limited to textbook descriptions.^1^ Here, we report such a case coexisting with nodular BCC.

An 80-year-old woman was referred to our hospital for excision of a lesion on her right forearm. The lesion had appeared 5 years previously and had gradually enlarged without any treatment. Clinical examination revealed a well-demarcated, elastic hard black nodule with some scales, measuring 14 × 14.5 mm, with good mobility over the underlying tissue, on the inner side of her right forearm (Fig. 1). Dermoscopy revealed large blue-gray ovoid nests, of multiple blue-gray globules, and shiny white areas. Under local anesthesia, the nodule was excised, and a full-thickness skin graft was performed. At a 4-month postoperative follow-up, there was no recurrence.

**Figure 1:**
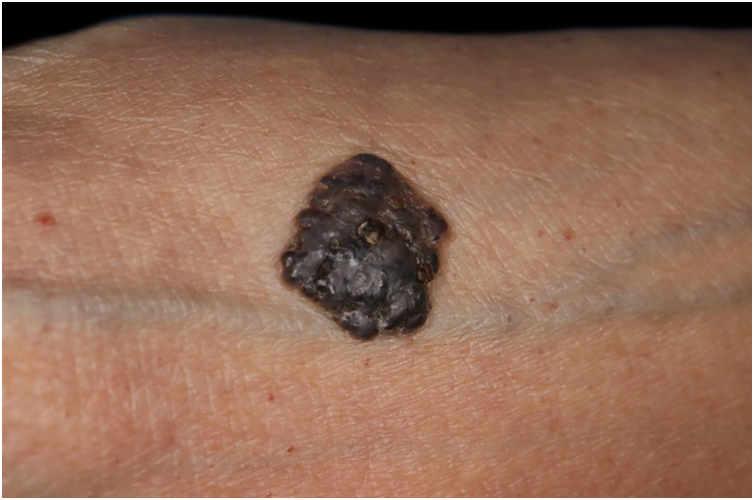
Clinical features of a well-demarcated, partially keratinized, elastic hard black nodule on the forearm.

Histopathological examination revealed a raised, protruding lesion with well-defined tumor nests of varying sizes within the dermis, some of which were continuous with the epidermis. Melanin granule deposition was prominent in the tumor nests and stroma (Fig. 2A). Nodular BCC was also identified. There were no findings indicative of differentiation into sebocytes or elements of hair papilla-like structure (Fig. 2B). The tumor nests exhibited a complex cord-like arrangement, referred to as a labyrinthine pattern. Mucinous spaces were observed between the cord-like tumor nests, with minimal presence of blood vessels and fibroblasts. The labyrinthine pattern was observed in approximately 70% of the entire tumor cell population (Fig. 2C‒D). Immunohistochemical findings showed diffuse BerEP4 expression in tumor cells (Fig. 3), and S-100 expression in approximately 5%. Adipophilin, GCDFP-15, CEA, EMA, vimentin, Melan A, and CK20 were not expressed in the tumor cells.

**Figure 2:**
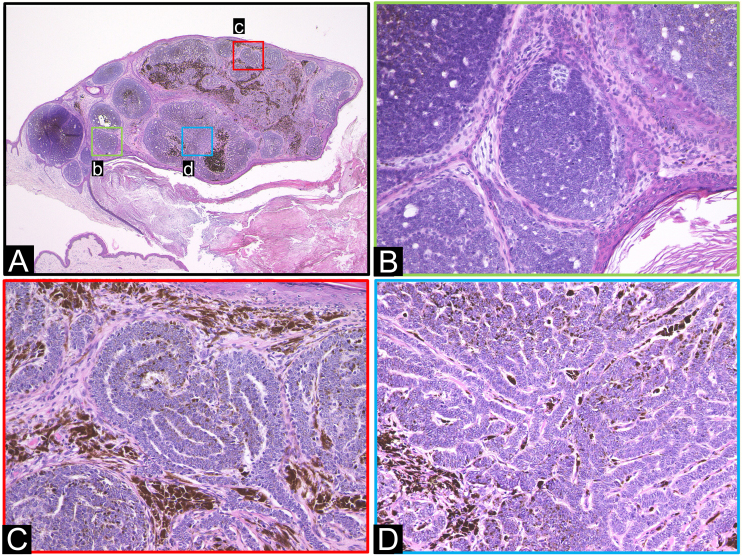
Histopathological features. (A) Protruding lesion with well-defined large and small tumor nests within the dermis. (B) Region of nodular BCC. (C‒D) Tumor cells exhibit a complex cord-like arrangement, forming a pattern described as labyrinthine. (Hematoxylin & eosin; A:×20, B:×200, C:×200, D:×200).

**Figure 3:**
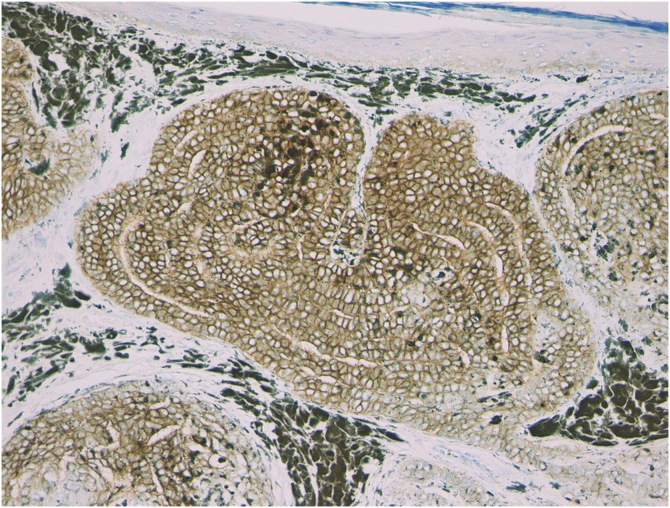
Immunohistochemical features showing diffuse immunoexpression of BerEP4. (×200).

The present case needs several histopathological differential diagnoses, including sebaceous adenoma, sebaceous carcinoma, trichoblastoma, and malignant melanoma.^2^ Sebaceous adenomas and carcinomas are known to have characteristic cellular arrangements, like rippled patterns, as shown in Table 1^3^; and distinguishing between them can sometimes be difficult. In this case, the results of immunohistochemistry revealed positive findings for Ber-EP4 but negative for S100, adipophilin, GCDFP-15, CEA, EMA, vimentin, and Melan A. Therefore, immunohistochemical staining facilitated differential diagnosis from other tumors. The absence of differentiation into sebocytes or elements of hair papilla-like structure, as well as the diffuse expression of BerEP4, led to the diagnosis of labyrinthine pattern BCC coexisting with nodular BCC. Trichoblastoma was excluded in that follicular germinative cells were absent.

**Table 1:** Differences in cellular arrangement.

**Labyrinthine Pattern**	Tumor cells exhibit a complex cord-like arrangement, forming a pattern described as labyrinthine. Edematous changes are observed between the tumor nests, but mesenchymal components such as blood vessels and fibroblasts are scarcely seen.
**Carcinoid-like Pattern**	Tumor cells are arranged in cords, ribbons, rosettes, and networks, displaying a histological appearance similar to carcinoid tumors. Mesenchymal components such as blood vessels and fibroblasts are interspersed between the tumor nests.
**Rippled Pattern**	Regions where nuclei are arranged in palisades alternate with anuclear regions in one direction, creating a rippled pattern. Cytoplasm without blood vessels is observed between the tumor nests.

While reports on the labyrinthine pattern in BCC are scarce, there have been 18 reported cases exhibiting carcinoid-like patterns and rippled patterns, all of which coexisted with nodular BCC, and in 15 out of the 18 cases, the lesions predominantly occurred on the face.^2^, ^4^, ^5^, ^6^, ^7^ Additionally, sebaceous adenomas and trichoblastomas with carcinoid-like patterns and rippled patterns have been reported to show apocrine gland differentiation,^8^, ^9^ but tubular structures in BCC with rippled patterns have not been reported to show signs of apocrine gland differentiation.^7^ This suggests that tumors exhibiting the same patterns may lose their differentiation potential in malignant BCC, in contrast to benign sebaceous adenomas and trichoblastomas.^7^ The coexistence of nodular BCC was also observed in the present case, but there were no findings indicating apocrine gland differentiation.

## Financial support

None declared.

## Authors’ contributions

Takehiro Nakamura: The study concept and design; data collection, or analysis and interpretation of data; writing of the manuscript or critical review of important intellectual content; data collection, analysis and interpretation; intellectual participation in the propaedeutic and/or therapeutic conduct of the studied cases; final approval of the final version of the manuscript.

Toshiyuki Yamamoto: The study concept and design; effective participation in the research guidance; critical review of the literature; final approval of the final version of the manuscript.

## Conflicts of interest

None.
